# Nanomaterial scaffolds for enzymatic polymer degradation: a tool to advance current biodegradation assessments of polymers in liquid formulation

**DOI:** 10.1186/s44331-025-00004-4

**Published:** 2025-08-01

**Authors:** Nick W. Johnson, Sheng Yan Jiang, Samuel B. H. Patterson, Trevor Hinchcliffe, Filipe Vilela, Humphrey H. P. Yiu

**Affiliations:** 1 Institute of Chemical Sciences, School of Engineering & Physical Sciences, Heriot-Watt University, Edinburgh, UK; 2 Impact Solutions Ltd., Impact Technology Centre, Fraser Road, Kirkton Campus, Livingston, UK

**Keywords:** Nanomaterials, Enzyme Immobilisation, Polymer Degradation, Biodegradation, Flow Chemistry

## Abstract

Polymers are used as an integral component in a wide range of liquid formulation products to improve formulation integrity and product performance. Due to environmental and regulatory pressures, it is necessary for the industry to shift away from petrochemically derived polymers to more sustainable and biodegradable products. However, current methods to analyse the biodegradation of polymers are time consuming and adapted to small molecules which is stifling innovation in this area. There is a requirement to re-envisage how the industry conducts biodegradability testing for polymers in liquid formulation (PLFs) from high-throughput screening methods at the preliminary stages of development to predictive modelling. Advancements in the use of nanomaterials as enzyme immobilisation scaffolds for polymer degradation could evolve how biodegradability testing is thought about and drive the PLF industry into a more sustainable future. This review highlights the current trends in the use of nanomaterials as enzyme immobilisation platforms and how this technology has been applied to the degradation of biodegradable PLFs.

## Introduction

Liquid formulation products represent a significant arm of the chemicals industry [1]. The addition of polymers is common practice amongst formulations to improve formulation integrity (i.e. rheology modification, as a stabilising agent) or act as an active ingredient in the formulation and are currently utilised within a diverse range of consumer-based and industrial products including personal care, paints, adhesives, agrochemicals and wastewater treatment industries [1, 2]. A report in 2021, collated by Royal Society of Chemistry (RSC) and the Centre for Process Innovation (CPI), estimated the total market value of polymers which are used in liquid formulation products to be up to $125 billion in 2021, and this value is expected to have increased since the publication of the study with the total global polymer market increasing at a compound annual growth rate (CAGR) of 4.8% [1, 3]. Polymers in liquid formulation (PLFs) can be defined as polymers which are liquid at the point of application and subsequently can be in either liquid or solid form after use [1]. The environmental impact of PLFs is often overlooked in public perception due to a lack of public and even scientific knowledge of their usage and fate. The vast majority of PLFs currently used in industry are chemically synthesised polymers from petrochemically derived monomers that include epoxy resins, polyurethanes, polysilicones, and polyacrylates [2]. Synthetic polymers are used by industry due to their advantageous chemical and physical properties including resistance to degradation, however, their use has raised concern due to their persistence in the environment and potential ecotoxicity [4, 5]. The 2021 RSC PLF report also estimated that the global PLF industry produces and uses 36.3 million metric tonnes of product annually with the vast majority being assumed to accumulate as waste and culminate in the natural environment and wastewater treatment facilities [1, 6]. To act upon this threat, the EU have tasked with the European Chemicals Agency (ECHA) with implementing new Registration, Evaluation, Authorisation and Restriction of Chemicals (REACH) legislation aimed at reducing the environmental impact of persistent polymers which could lead to the formation of problematic microplastics [7, 8]. To clarify the definition of a microplastic for this review, it defines a non-biodegradable synthetic polymer below 5 mm in size which are insoluble in an aqueous media [9, 10]. This legislation is being implemented gradually over a 12-year period and from October 2027, the use of microplastics or polymers which have the potential of forming microplastics will be restricted in liquid formulation products [8]. This change in regulation forces the PLF industry to reduce its reliance on petrochemical feedstocks and become more sustainable [2].

**Fig. 1:**
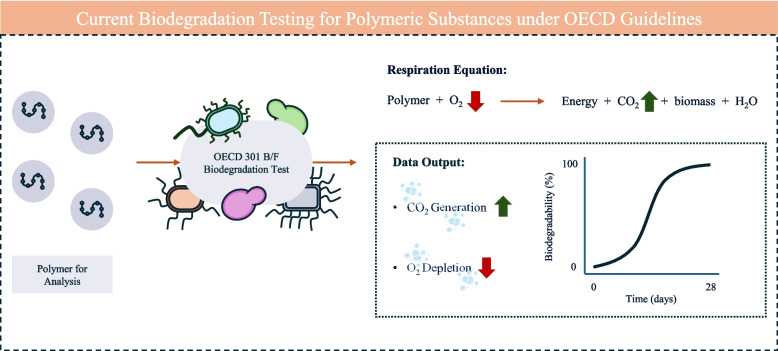
Current OECD standard testing guidelines for the biodegradation of polymeric substances. Guidelines for OECD 301 C (modified Sturm test measuring CO_2_ evolution) and 301 F (manometric respirometry test measuring O_2_ consumption) are the most widely used industry for polymers over a minimum of 28 days and a maximum of 2 years. Biodegradability tests (OECD 301) are prepared in aqueous media inoculated with an activated wastewater treatment culture and analysed at periodic time points to assess ready and inherent biodegradability

To address sustainability, industry has focussed on replacing current polymers in use with biodegradable alternatives [11–15]. The International Union of Pure and Applied Chemistry (IUPAC) defines biodegradable polymers as ‘*polymeric substances which are susceptible to breakdown by biological processes’* [16]. Biodegradation requires the participation of living organisms though polymer degradation can proceed either in vivo and/or in vitro [17]. In the natural environment, the physical and chemical properties of the polymer can be altered by abiotic chemical processes and weathering though do not participate in the biodegradation process [18]. To clarify, this contrasts to i) bio-based polymers in which the polymer is obtained from a biological source, and ii) bio-derived in which the monomers or chemical feedstock is extracted from a biological source and polymerised chemically [1, 4]. Microorganisms typically utilise the constituent monomers of the polymer as a carbon source for cell function and replication, therefore, reduce environmental persistence compared to synthetic alternatives. Various naturally occurring biodegradable polymers are already utilised within the market including polysaccharides, polyesters and polyamides [19–24]. One of the key challenges to replace existing PLFs with biodegradable alternatives is to maintain and/or improve overall performance of the consumer product. However, the development of the biodegradable PLFs is impeded by current methods to determine whether a product is biodegradable. Current standard biodegradation tests within the International Organization for Standardization (ISO) framework are carried out under the Organisation for Economic Co-operation and Development (OECD) guidelines for a CO_2_ evolution (modified Sturm) test (OECD 301B) or a manometric respirometry test (OECD 301 F) to assess ready biodegradability [25]. Under these guidelines, tests can take from 28 days up to 2 years to complete with the only data output being either a measurement of CO_2_ released or dissolved O_2_/organic carbon content (Fig. 1) [7, 26, 27]. Aside from the biodegradation testing being costly and low-throughput, polymers are subject to the same standard biodegradation tests which have been designed for small molecules, including pharmaceuticals that are subject to stricter environmental fate regulations, and undergo a different biological degradation to polymeric substances [6, 28, 29]. To drive innovation in this area of research, the RSC along with many industries leaders have highlighted that not only does the current biodegradability standard testing of polymers need to be re-addressed but alternative testing methods are required to run alongside current testing methods to facilitate development [1, 2, 27]. To this end, there is an opportunity for the development of in vitro polymer biodegradation studies alongside current testing methods. Furthermore, recent advancements utilising nanomaterials as enzyme immobilisation scaffolds (or carriers) to further improve enzyme-dependent PLF biodegradation could unlock the key to reliable and robust testing methods required by industry [30, 31].

Enzyme immobilisation is a widely applied technique within organic chemistry and biotechnology for the synthesis of key pharmaceutical intermediates, as well as having varied application in the food, medical and water treatment industries [30, 32–35]. The advancement in this technology has provided a platform for use of enzymatic processes in industry to widen and make them an economically viable option by improvements in enzyme activity and reusability, through improving the enzymes stability and resistance to deactivation [36]. Conventional immobilisation techniques rely either on i) a carrier support which is either bound via electrostatic or covalent linkages to bind the enzyme to the surface of the carrier, ii) entrapment of the enzyme within a solid or gel matrix, or iii) carrier free cross-linked enzyme aggregates [35, 36]. The optimal choice of immobilisation technique and/or carriers is often application dependent but typically aims to improve overall performance of the process and protect the enzyme from environmental changes [35]. Though, bulk organic and inorganic materials are commonly used due to cost and availability, model immobilisation supports seek to take advantage of a porous support matrix including mesoporous silica and carbon based materials (pore size typically below 2–50 nm, as defined by IUPAC) to maximise the protective environment whilst providing a high surface area for protein binding [37–39]. However, the hydrodynamic radius of high molecular substrates are similar to or exceed the pore size of nanoporous supports, negatively impacting retained enzyme activity due to diffusion limitations [40, 41]. To overcome this challenge, there have been recent advancements in the use of nanomaterials as immobilisation scaffolds for enzymatic polymer degradation, in addition to polymer synthesis though examples are more limited [42–44]. Nanomaterials offer a high surface area with enhanced enzyme loading and a higher degree of easily accessible surface facing enzymes compared to conventional porous scaffolds, reducing the diffusion limitation of high molecular weight substrates/products [41, 45]. Due to the relevance of these advancements towards the biodegradable PLF market, this review will highlight the recent examples of the use of nanomaterials as an enzyme immobilisation scaffold for polymer degradation. In particular, this review will focus mainly on the major advantages of using different nanomaterials in the degradation of polysaccharides, a major class of biodegradable PLF, as well as additional hydrolytic enzymes important for the degradation of polyesters and polyamides and redox-dependent oxidoreductases important for the degradation of lignin [46]. Finally, the review will demonstrate how this technology could be reapplied to the biodegradability testing market and aid the development of novel biodegradable polymers.

### Microbial polymer degradation

The manner in which nature degrades naturally occurring polymers using enzymes has been studied since Payne and Persoz identified the first diastase enzyme in the mid-nineteenth century for the breakdown of the polysaccharide, starch, into maltose [47]. Subsequent studies over the past century have led to gaining a deeper understanding into the enzymatic mechanisms of how polymers are broken down in natural environmental settings being established [46, 48, 49]. The term biodegradation refers to the ‘*degradation resulting from cell activity*’ [50]. Within this umbrella term, there are four stages which encompass the polymer biodegradation process; i) biodeterioration which refers to the modification of the polymer’s chemical, physical, and mechanical properties from a cellular-derived process and/or entity; including enzymatic, product of a metabolic process and biofilm formation, ii) biofragmentation which refers to biotic polymer lysis, typically carried out enzymatically, decreasing the molecular weight of the polymer to oligomers and monomers to allow for cellular uptake, iii) microbial assimilation, and subsequently iv) mineralisation allowing for the organic polymer to be converted into CO_2_ and H_2_O, in aerobic conditions, and additionally methane if the process is anaerobic [18, 48, 49]. Although the term biodegradation only relates to cell-mediated degradation, biodegradation is also influenced by a number of abiotic factors in the natural environment including chemical, photochemical and physical processes which cause polymer deterioration and fragmentation and aid the biodegradation process [18, 49]. The major classes of enzymes responsible for enzymatic polymer fragmentation are oxidoreductases, including lignin peroxidase which conduct redox chemistry, and hydrolases, though the latter are by far the most common [46]. The reaction mechanisms of each hydrolase vary depending on the specific enzyme, though all depend on a water molecule to act as a nucleophile [51, 52]. There is an emerging field of research within synthetic biology in the identification and engineering of evolved enzymes for the enzymatic degradation of synthetic polymers [53, 54]. Though promising, these processes seek to establish enzymatic reactivity not seen in nature and not a direct representation of the natural environment, thus, are beyond the scope of this review. This review aims to focus on the enzymatic degradation of polymers which are currently utilised within the PLF market and are biodegradable in the natural environment.

**Fig. 2:**
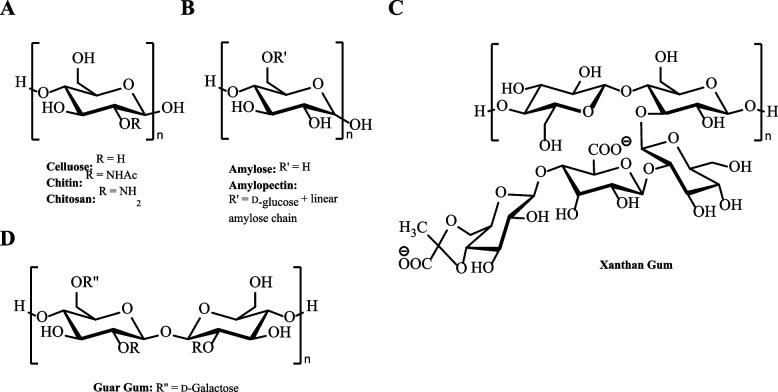
Chemical structures of the polysaccharides; **A** cellulose, chitin, and chitosan, **B** amylose and amylopectin, **C** Xanthan gum, and **D** Guar gum

A major class of biodegradable polymer currently used in the PLF industry are polysaccharides [2, 27]. Polysaccharides, with a general formula of (CH_2_O)_x_, are naturally occurring bio-macromolecules composed of single sugar (monosaccharide) units [55]. Cellulose, for example, is a structurally important biopolymer in the formation of cell walls in plants and is composed of a linear chain of repeating β-glucose bonded via β−1,4 glycosidic linkages (Fig. 2A) [56]. The biopolymer is readily biodegradable with a combination of endo-β−1,4-glucanase, cellobiohydrolase, and β−1,4-glucosidase enzymes having evolved for the complete hydrolysis of cellulose to monomeric units [57]. Though, native forms of cellulose have application within the PLF industry, the extensive hydrogen bond network, low water solubility and high rate of biodegradation of cellulose can limit its function [58, 59]. To address this, the hydroxyl groups of the glucose units can be further chemically modified to improve the polymers properties for application but also the rate of biodegradation can be tuned to improve its biostability [59, 60]. For example, cellulose modified with hydroxypropyl methylcellulose using propylene oxide and methyl chloride can vastly improve water solubility and viscoelasticity of cellulose and expand its application in use as a film former in agriculture and rheology modifier and emulsifier in the pharmaceutical industry [61–64]. However, the modification of the physical and chemical properties is dependent on the degree of substitution (DoS) and can be further tuned to cater for specific applications [2, 27, 60]. The choice of functional group(s) conjugated to cellulose is vital for desired function. The conjugation of charged moieties can target interactions with charged molecules which, among others, has found application as a flocculant to remove waste materials in wastewater treatment [65]. As a result, there now is a large range of cellulose-based derivatives for use on the PLF market including carboxymethylcellulose, cellulose acetate, and methylcellulose [66].

Apart from chemical modification, the use of more structurally diverse polysaccharides has been employed in the PLF market. Starch is another common example, which chemically differs from cellulose by altering β-glucose to α-glucose, bonded via α−1,4 glycosidic linkages to form linear amylose that is enzymatically hydrolysed by α-amylase (Fig. 2B) [67, 68]. However, starch can also form α−1,6 glycosidic bonds to create highly branched polymers, amylopectin, which vastly alters the chemical and mechanical properties in terms of water solubility, hydrogen bonding and tensile strength [68]. Due to this branching, the chemical structure is more accessible to enzymatic degradation from α-amylase and β-amylase and generally considered to have a higher degree of biodegradability than cellulose [67]. Starch is, therefore, used heavily within the food industry as a rheology modifier due its increased digestibility compared to cellulose, as well as, application in personal care formulations providing superior sensory properties [68, 69]. Additional water-soluble non-starch-based polysaccharides are increasingly being used as PLFs [70]. Hydrocolloids, as they are commonly named, include Xanthan and Guar gums which are extracted from the Gram-negative bacterium *Xanthomonas campestris* and the legume *Cyamopsis tetragonoloba*, respectively, and exhibit pseudoplastic properties without chemical modification due to shear thinning behaviour [71–73]. This enables the polymer to be used at a lower working concentration whilst displaying high thermal and pH stability in formulation [70, 71, 73–75]. Xanthan and Guar gums have branched structures with a polymer backbone consisting of D-glucose and D-mannose monomers linked via β−1,4 glycosidic bonds (Fig. 2C and D) [11, 76]. Although micro(organisms) have evolved for the specific utilisation of Xanthan and Guar gums, the glycosidic bonds which the polymers are composed of are ubiquitous in nature and enzymes have displayed promiscuity towards these substrates [11, 76]. The exact mechanisms of degradation are unknown but, fungi such as *Trichoderma reesei* are thought to synthesise high levels of β−1,4-mannanase, capable of hydrolysing the polymer backbone of Guar gum, and in the instance of Xanthan gum, a combination of native fungal and bacterial α- and β−1,4-mannosidase, β−1,4-glucosidase, and endo-β−1,4-glucanase enzymes are thought to hydrolyse the polymer to its constituent monomers and, as a result, both polymers exhibit ready biodegradability [11, 76–78].

Beyond polysaccharide chemistry, naturally occurring and bio-derived polyesters are employed by the PLF market [79, 80]. The main sub-classes of enzymes responsible for ester hydrolysis, typically, are known to be esterases, cutinases, and lipases [81]. These enzymes form a part of a larger class of serine hydrolases which are a naturally abundant enzymes capable of hydrolysing ester, amide, and thioester bonds, relying on a catalytic triad within the active site, consisting of a nucleophilic serine, histidine and aspartic acid (though in some instances, particularly within proteases, serine can be replaced with cysteine) [82, 83]. The substrate specificity varies drastically among enzyme homologs capable of ester hydrolysis. For example, lipases require non-polar lipophilic compounds to activate the ‘lid’ like structure that results in conformational change and the active site being accessible to the substrate [84]. Lipases, consequently, have found industrial applicability being tolerant to high levels of organic solvent as well as accepting a large number of substrates [51]. In contrast, esterases tend to be active towards small water-soluble compounds though polymer degradation has been widely demonstrated [85]. As an example, polyhydroxyalkanoate (PHA) depolymerases are naturally abundant carboxylesterases, commonly found among bacteria, that have evolved to depolymerise PHA derivatives efficiently as an energy source [86]. Polymer biodegradation rate is dependent on many factors including polymer structure, molecular weight, and crystallinity [87]. For example, the enzymatic hydrolysis of polylactide (PLA) is known, however, the rigid structure of PLA restricts enzyme accessibility to the polymer ends which decreases the rate of enzymatic hydrolysis [88, 89]. However, for the purpose of this review, PLA is not considered biodegradable unless demonstrated, as high molecular weight PLA is not degraded by native microorganisms and relies on abiotic hydrolysis to fragment the polymer into biodegradable products, therefore considered hydrolytically degradable [90]. Nonetheless, due to the high promiscuity of many serine hydrolases many synthetic polyesters have been demonstrated to be readily biodegradable, including polycaprolactone (PCL), many of which can be synthesised via enzymatic methods [91]. Additionally, the advancements in synthetic biodegradable polyesters have allowed for the further development block co-polymers to further tune polymer properties and biodegradability [92, 93].

The development of novel biodegradable polymers for the PLF market is gaining momentum due to efforts to improve sustainability and impending regulatory changes [1, 6]. As a result, biodegradability needs to be determined early in the product development process and screened to ascertain whether the product meets standard requirements for further development. Current standard biodegradation tests do not facilitate this development, relying on the slow and variable growth of native microbial consortia [94, 95]. To adapt to this evolving market, novel in vitro enzymatic degradation testing methods need to be established to improve both through-put, as well as data output, by reducing the variability of cell growth and providing a specific environment for polymer degradation. By combining in vitro testing with current enzyme immobilisation technology to enhance enzyme stability and reusability, this would allow for the creation of a robust testing platform capable of analysing sequential polymer samples required by industry to manage an increased market demand. Though numerous immobilisation platforms have been used, nanomaterials have emerged as the benchmark scaffold for enzymatic polymer degradation due to their advantageous characteristics, which the remaining part of this review will discuss [96].

### Use of nanomaterial scaffolds for enzyme immobilisation

Nanomaterials make a key contribution in everyday life and drive innovation within key industries such as electronics, construction, and medical devices owing to their unique and tuneable properties including mechanical strength, conductivity and magnetism [97]. In recent years, the use of nanoparticles/nanomaterials as an enzyme immobilisation scaffold has been realised, often showing superior characteristics to bulk materials including a higher surface area to volume ratio, enhanced activity, and improved reusability [98, 99]. A nanomaterial can be defined as a structure with one or more dimensions, with one of the dimensions measuring less than 100 nm, whereas a 0-dimensional nanostructure can be defined as a nanoparticle [97]. Nanomaterials can either be composed of organic or inorganic compounds, including silica, carbon, and metal oxides and are synthesised either via a top-down or bottom-up approach which fabricate the nanomaterial from either the bulk material or individual atoms, respectively, with the latter approach allowing for more precise control over size and shape with an improvement in overall homogeneity in chemical composition [98]. Immobilised enzymes are bonded to the nanomaterial support either through non-covalent or covalent interactions [99]. Enzymes bound to immobilisation supports through Van der Waals or hydrophobic interactions are widespread, though the strength of the interaction is relatively weak, and the enzyme is prone to leaching [98, 100]. Therefore, standard practice for enzyme immobilisation, particularly within inert inorganic nanomaterial supports, is to establish stronger covalent or ionic bonds which requires further functionalisation steps unless incorporated during nanomaterial synthesis (co-condensation) [97, 101]. Generally, this is achieved through an initial grafting step to introduce a functional handle which can directly bind to the enzyme or is selected to react with an appropriate cross linker [102]. Alkyl trialkoxysilanes, such as aminopropyl trialkoxysilanes, (RO)_3_Si–(CH_2_)_3_–NH_2_, are commonly used due to their cost and commercial availability, in addition to phosphonic acid, mercaptans, and catechol although the functional group can be tuned [102, 103]. A cross linker may be required to target the nucleophilic side chains of non-catalytic lysine and cysteine surface residues for bioconjugation but also to allow the linker to be tuned to provide an optimal distance between protein and support to maintain protein function [103, 104]. Various bioconjugation linkers are employed including thionyl chloride, cyanuric chloride, cyanogen bromide, sulfonyl/metal/acyl halides, carbodiimides, but the use of either glutaraldehyde or succinimidyl 4-(*N*-maleimidomethyl)cyclohexane-1-carboxylates derivatives which target surface lysine and cysteine residues, respectively, are amongst the most prevalent [103]. However, the choice of chemicals for functionalisation (grafting and cross linking) is entirely dependent on the specific application and multiple factors need to be considered including linker length, amino acid residues on the protein surface, cost, and bond stability, though, one key common feature is that the enzyme binding step tends to be carried out at around room temperature (4–37 °C) to preserve the integrity and activity of the enzymes [105, 106]. Though significant research efforts in enzyme immobilisation are focussed on medical applications and the sustainable production of bulk/specialised chemicals, over the past decade, immobilisation on a nanomaterial supports have gained interest for polymer degradation primarily to produce biofuels and renewable feedstocks for industrial biotechnology through recycling waste materials such as lignocellulosic biomass [30, 42]. Through the development of this technology, there is significant potential to expand its application into enzymatic fragmentation analysis, though further research is required. Therefore, the rest of this review will focus on recent advances in polymer degradation using enzymes immobilised on nanomaterials supports. Nanomaterials are categorised as either 0, 1, 2, or 3-dimensional materials with each dimension providing specific characteristics advantageous to enzyme immobilisation. The following sections will divide nanomaterials based on dimension discussing the advantages and disadvantages and consider how chemical composition impacts the properties of each nanomaterial (Table 1).

**Table 1:** Examples of nanomaterials used as enzyme immobilisation scaffolds. Each enzyme that has been highlighted has been recognised for its importance in the biodegradable PLF market

Target biodegradable polymer	Chemical structure	Nanomaterial (OD, 1D, 2D, 3D)	Nanomaterial composition	Immobilised enzyme	Reference
Cellulose	D-glucose bonded via β-(1,4)-glycosidic bonds (linear).	0D	Fe_3_O_4_ NP	Cellulase (combination of endo-β-1,4-glucanases, cellobiohydrolase, β-1,4-glucosidase)	[107]
		‘’	Fe_3_O_4_/Chitosan NP	‘’	[108]
		‘’	Fe_3_O_4_/Chitosan/GO NP	‘’	[109]
		1D	ZnO NW	β-1,4-glucosidase	[110]^a,b^
		2D	Fe_3_O_4_/Halloysite NT	‘’	[111]
		3D	Mesoporous Silica Nanowrinkles	Cellulase with β-1,4-glucosidase	[112]
		‘’	‘’	Cellulase	[40]
		‘’	Ceramic cordierite monoliths/ Mesoporous Silica Nanowrinkles	β-1,4-glucosidase	[113]^a,b^
Starch	D-glucose bonded via α-(1,4)-glycosidic bonds (linear version - amylose). Amylopectin contains additional α-(1,6)-glycosidic bonds (branched version).	0D	Ag NP	α-amylase	[114]
		‘’	Au NP	‘’	[115]
		‘’	‘’	‘’	[116]
		‘’	ZnFe_2_O_4_ NP	‘’	[117]^a^
		1D	CuO NW	‘’	[118]
		‘’	Electrospun poly(styrene-*co*-maleic anhydride) nanofibre	α-amylase (and protease co-immbolisation)	[119]
		2D	GO NS	β-amylase	[120]
			MoS_2_ NS	‘’	[121]
			ZnO NS	α-amylase	[122]
Chitin/Chitosan	*N*-acetylglucosamine bonded via β-(1,4)-glycosidic bonds (linear).	OD	ZnO NP	Chitinase	[123]
		‘’	‘’	‘’	[124]
Polyesters	Bonded via ester bonds.	0D	ZnFe_2_O_4_ NP	Lipase	[117]^a^
		‘’	MgNF	‘’	[125]^a^
		‘’	Fe_3_O_4_ NP	‘’	[126]^a^
		‘’	CoFe_2_O_4_	‘’	[127]^a^
		‘’	Halloysite NT	‘’	[128]^a^
		1D	SWCNT	‘’	[129]^a,b^
		3D	MSN/Fe_3_O_4_	‘’	[130]^a,b^
		‘’	Fe_3_O_4_/GO	‘’	[131]^a^
Polycaprolactone	Composed of 6-hydroxyhexanoic acid monomers (linear). (Typically, synthesis via ring opening polymerisation of ε-caprolactone).	2D	GO NS	‘’	‘’^a^
		‘’	GO/Fe_3_O_4_ NS	‘’	‘’^a^
		‘’	GO NS	‘’	[132]^a^
		3D	HMSN shell/Fe_3_O_4_ core	‘’	[133]
		‘’	‘’	Cutinase	‘’
Poly(*L*-lactide)^c^	Composed of L-lactic acid monomers (linear).	3D	SBA-15	Proteinase K	[89]
Lignin	Composed mainly of sinapyl alcohol,* p*-coumaryl alcohol, coniferyl alcohol monomers bonded via C-C, ether and ester bonds.	0D	Fe_3_O_4_/SiO_2_/polydopamine	Lignin peroxidase	[134]^a^
		1D	SWCNT	Lignin peroxidase	[135]^a^
		‘’	SWCNT	Lignin peroxidase, Manganese peroxidase, and Laccase	[136]
		2D	Halloysite NT	Laccase	[128]^a^
Proteins	Amino acids bonded via amide bonds.	0D	ZnFe_2_O_4_	Protease	[117]^a^
		1D	Electrospun poly(styrene-*co*-maleic anhydride) nanofibre	Protease (and α-amylase co-immobilisation)	[119]
		2D	GO NS	Protease	[137]^a^
		3D	CuSO_4_/Fe_3_O_4_ NP nanoflower	Collagenase	[138]

^a^Polymer degradation not been observed experimentally

^b^Immobilisaed enzyme system has been utilised in continuous flow

^c^Poly(*L*-lactide) is not biodegradable unless demonstrated, otherwise is considered as hydrolytically degradable

## Polymer degradation using enzymes immobilised on nanomaterials

### 0-D Nanomaterials

**Fig. 3:**
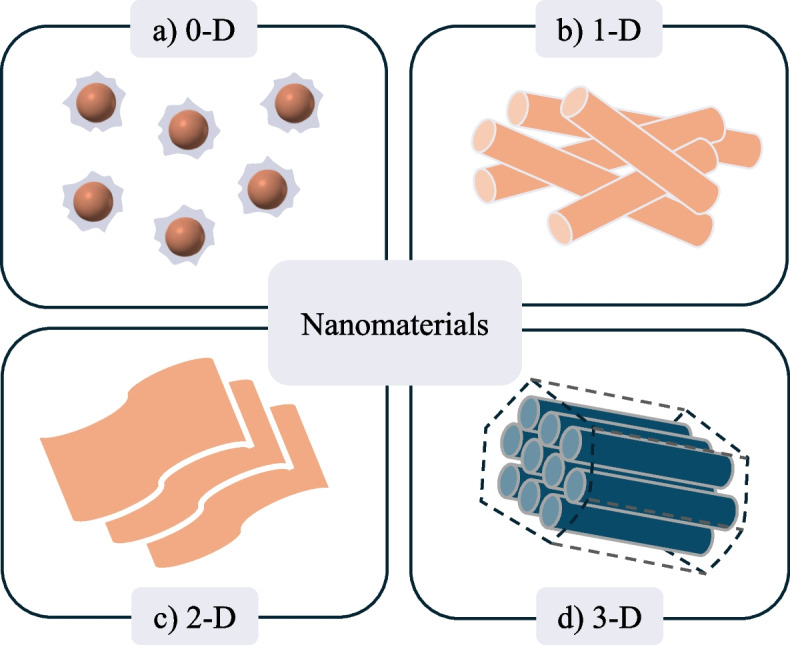
Representative structures of the different nanomaterials employed for enzyme immobilisation. **a** 0-D nanomaterials e.g. nanoparticles, **b** 1-D nanomaterials e.g. nanorods and nanowires, **c** 2-D nanomaterials e.g. nanosheets, **d** 3-D nanomaterials e.g. hollow mesoporous silica nanoparticles

Nanoparticles (NPs) are 0-D nanomaterials technically ranging from 1–100 nm in size, though the cutoff is widely regarded as below 300 nm (Fig. 3A) [97, 139]. NPs can be composed of either inorganic and organic materials, though, silica (SiO_2_), metal oxides, polysaccharide (e.g., dextran, chitosan) and their composites are amongst the most popular nanoparticle materials for enzyme immobilisation [140]. Generally, enzyme immobilisation will proceed on the surface of the NP which provides a high surface area to volume ratio resulting in high enzyme loading and a potential solution to the diffusion limitations exhibited when using nanoporous materials [41, 141]. Due to their ease of synthesis and cost, metal oxide NPs have extensively been utilised as enzyme immobilisation platforms [142]. Additionally, biology has evolved to be able to prepare metal nanoparticles through the use of membrane proteins and metabolically produced reducing agents to reduce numerous metal ions including gold, palladium, and nickel demonstrating their biocompatibility and low toxicity [143–145]. For instance, zinc oxide (ZnO) NPs have recently been utilised as a biosensor for pathogenic fungi on papaya peel [123]. The NP acted in a dual capacity as an enzyme support, as well as an electrochemical inducer, due to the conductive properties of ZnO. The group targeted the enzymatic degradation of the polysaccharide, chitin, a key structural component in fungal cell walls, as the basis for the biosensor, measuring changes in electrical frequency as a method to detect fungal contamination. Chitin is a linear polysaccharide, composed of *N*-acetyl glucosamine monomers, which is natively hydrolysed by chitinase enzymes (Fig. 2A) [146]. Subsequently, the group directly immobilised a chitinase from *Trichoderma spp.* onto ZnO NPs, prepared via precipitation, before being place into contact with a gold electrode. A change in electrical frequency in the papaya displaying fungal growth confirmed enzyme activity as well as the biosensors applicability. In a separate study, a chitinase from the gram-positive bacterium, *Lactobacillus coryniformis* 3N11, was isolated and immobilised also on ZnO NPs. The immobilised enzyme was observed on the surface of the NP and displayed an improvement in enzyme activity compared to the free enzyme. The ZnO-chitinase NP could subsequently be employed as an insecticide to protect corn crops against insects with a chitin exoskeleton [124]. Both ZnO bioconjugates highlighted were prepared via physical adsorption, relying on relatively weak non-specific intermolecular forces such as van der Waals forces. In the context of this review, direct adsorption onto the NP can be a highly effective strategy due to the high proportion of polymer-degrading enzymes present in the growth media of appropriate cell cultures, as the cell seeks to break down the high molecular substrates for assimilation extracellularly [147, 148]. As a result, the effective concentration of the desired enzymes increases in the growth media of non-lysed cell cultures whilst undesired proteins largely remain in the cell interior, reducing the requirement for purification. When direct immobilisation to the NP is desired, a stronger bond can be established with the use of silver and gold NPs which exhibit a high binding affinity to thiol groups of cysteine, forming a stable bond partially covalent in nature [149]. When Ernest et al. immobilised a porcine-derived α-amylase on to the surface of Ag NPs, the researchers hypothesised that the terminal aldehyde groups of starch reduced silver nitrate to atomic silver which could subsequently react with thiol groups of cysteine on the surface of α-amylase. [114] This resulted in a strong interaction between enzyme and support whilst improving starch degradation 1.5-fold, compared to the free enzyme.

Metal and metal oxide NPs exhibit low stability in aqueous media and have a tendency to aggregate [115, 116]. In order to control aggregation, a coating can be used to protect the NPs surface [150]. The composition of the coating can either be a metal, i.e. gold, or an (in)organic polymer including silica, polysaccharides, and synthetic polymers such as polymethacrylate. In this instance, magnetite, Fe_3_O_4_, is an extensively utilised nanoparticle in enzyme immobilisation due to its magnetic properties [107, 151]. This is advantageous for separating the immobilised enzyme from the spent media, and in turn, reuse of the enzyme, as well as for enzyme isolation and purification from growth media [100]. However, magnetite is easily oxidised and coating the Fe_3_O_4_ NPs can provide protection [152]. There are now many important examples of enzyme immobilisation on coated Fe_3_O_4_ NPs for PLF degradation, including the immobilisation of chitosanases, α/β-amylases, lipases, proteases, and cellulases [108, 117, 153–157]. For the latter, the enzymatic degradation of cellulose requires three different specialised enzymes (β−1,4-glucanases, cellobiohydrolase, β−1,4-glucosidase) and procedurally it is common to co-immobilise all three enzymes using a commercially available mixture [57]. Relative activities of each enzyme are subsequently tested using three different substrates; filter paper, carboxymethylcellulose, and *p*-nitrophenyl-β-d-glucopyranoside to test the total cellulase activity and the activities of β−1,4-glucanase and β-glucosidase, respectively [108, 158–160]. One of the major drawbacks of enzyme immobilisation is that overall activity is reduced compared to the free enzyme, and this was observed when Sánchez-Ramírez et al. co-immobilised a cellulose mixture from *T. reesei* onto Fe_3_O_4_ NPs coated with chitosan, which reduced total cellulose activity by 59% compared to when the free enzyme was tested [108, 161]. The reasons for a reduction in activity are case-specific and due to a number of reasons including diffusion, structural changes to the protein, access to the active site being hindered, and increased aggregation [162, 163]. In addition, the co-immobilisation of an enzyme mixture to a NP surface can be difficult to control as each enzyme exhibit different binding affinities and energy barriers to bonding affecting the uniform distribution of each enzyme [45, 164]. Though, immobilised enzymes typically exhibit higher stability at high temperatures and improved storage at low temperatures (4 °C) compared to the free enzyme and, in this instance, relative activity remained above 80% after 15 cycles of use. The use of chitosan as a coating provides a functional handle (-NH_2_) on the surface of the NP for covalent binding of enzymes whilst also being a naturally derived feedstock [165]. The chitosan is not hydrolysed in the process due to the specificity of the enzymes required for enzymatic cellulose degradation. However, the choice of coating is important, especially when employing readily biodegradable polymers, to ensure stability of the NP-enzyme complex. Aside from providing a functional handle and protection, NP coatings can also improve mechanical strength and enzyme loading. This has been demonstrated when using graphene oxide (GO) as a coating for Fe_3_O_4_ NPs, the surface area of the nanoparticle increased 2.2-fold and subsequently was employed as a Fe_3_O_4_/GO/chitosan nanocomposite used to immobilise the cellulase mixture from the fungus *Aspergillus niger* for biofuel production [109].

Aside from saccharification, the application of NPs as an enzyme immobilisation platform has been translated to further enzymes important for the study of PLF enzymatic degradation. For example, Fe_3_O_4_ NPs coated with GO have also been employed to the immobilisation of lipase B from *Candida antarctica* (CALB) [131]. This enzyme is of high importance to the chemical industry exhibiting a high substrate scope for both lipophilic and hydrophilic compounds and a tolerance towards high levels of organic solvent [166]. Important for this review, CALB been demonstrated to hydrolyse synthetic polyesters, including PCL, leading this homolog to become a model enzyme in polyester degradation. However, *C. antarctica* is a geographically limited fungus, originating from sediment in an Antarctic Lake [167]. While its application in biodegradation studies alone is restricted, CALB tests as a case study in how immobilising a lipase on a different nanomaterial can affect enzymatic polymer degradation in vitro to be able to better inform the future development of immobilised lipases. Encouragingly, there are examples of other lipases being immobilised onto NPs such as lipases from the Gram-positive bacteria *Bacillus thermoamylovorans* and *Bacillus subtilis* immobilised on to magnesium ferrite (MgNF) and Fe_3_O_4_ respectively, and a lipase from the fungus *Rhizopus oryzae* immobilised on silica-coated cobalt ferrite (CoFe_2_O_4_) NPs, which represent genera widely represented in the environment, though in these instances, their polymer degrading capabilities need to be studied [125–127]. Additionally, when testing in vitro, further focus is required to ascertain whether enzymatic polymer degradation mimics biofragmentation in the natural environment. To this end, researchers can also take advantage of NPs co-immobilisation efficiency because environmental degradation is not usually dependent on one microorganism but a microbial consortium which synthesise numerous enzyme homologs capable of hydrolysing the polymer [168].

Beyond hydrolysis, oxidoreductases are another family of enzymes important for polymer degradation [91]. Lignin is a naturally abundant aromatic rich polymer found in cell plant walls, consisting of coumaryl, coniferyl, and syringyl alcohol monomers, bonded via ether or carbon–carbon bonds [169]. The polymer has found application as a biodegradable PLF including in sunscreen, to improve sun protection levels, and as a water-proofing agent in coatings and adhesives [169, 170]. Enzymatic lignin degradation requires the iron-dependent lignin peroxidase, manganese peroxidase, or laccase to break the C–O–C and C–C bonds within lignin’s chemical structure releasing secondary phenolic pollutants [171, 172]. Much of the focus of in this research area is on the environmental remediation of these organic pollutants, but crucially methods have been discovered to analyse the fate of lignin-derived products after enzymatic degradation [173]. For example, the degradation of multiple phenolic compounds including phenanthrene, benzo(a)pyrene, and dibutyl phthalate has been studied using a lignin peroxidase, from the yeast *Pichia methanolica*, covalently immobilised onto a Fe_3_O_4_/SiO_2_/polydopamine NP [134]. Even though this research primarily focussed on wastewater remediation, it can also be envisioned that lignin fragmentation and depolymerisation could also be tested using this system. As in this study, the NPs could be removed from the reaction sample via a magnet and degradation products could be analysed via liquid chromatography-mass spectrometry (LC–MS) with minimal sample preparation. A key consideration of using oxidoreductases over hydrolases is that the enzyme proceeds through a radical mechanism, therefore, requiring an electron donor [171]. In the environment, this is provided via microbial metabolism, though for in vitro analysis an electron supply via a co-substrate i.e. hydrogen peroxide (H_2_O_2_) is required [174]. Though this section has described the use and advantages of NPs as enzyme immobilisation supports, NPs often are limited when enhanced electrical conductivity is required. In this instance, dimensional nanomaterials have been demonstrated to show superior conductive properties with improved tunability for enzyme immobilisation, which the next section will introduce [175].

### 1-D Nanomaterials

A one-dimensional nanomaterial differs from NPs in that only 2 of the dimensions remain at nanoscale (< 100 nm), allowing for the formation of tube or wire-like structures (Fig. 3B) [176]. These 1-D nanomaterials similarly require surface functionalisation for covalent enzyme immobilisation, however, due to their availability and cost, they are less common for use as an enzyme carrier than 0-D nanomaterials. Nonetheless, there have been numerous recent examples testing enzymatic polymer fragmentation using a 1-D nanomaterial as an enzyme scaffold. The most common examples are carbon nanotubes (NT), metal nanowires (NW), and organic polymer nanofibers (NF) [177]. Aside from improving stability and reusability, 1-D nanomaterials tend to exhibit improved enzyme performance compared to planar enzymes supports [178]. Similar to NPs, the small diameter of the nanomaterial provides a surface with a high radius of curvature [135]. This can result in a higher loading of active enzymes by reducing deactivating surface-protein and protein–protein interactions triggering conformational change [178, 179]. This was observed when lignin peroxidases from two fungus isolates, *Pleurotus ostreatus* (PLO9) and *Ganoderma lucidum*(GRM117), were immobilised by adsorption on single walled carbon nanotubes (SWCNT). An 18-fold and 28-fold increase in specific activity was observed, respectively, when compared to the free lignin peroxidase [135]. This increase in activity is reflected in other enzymes immobilised by adsorption on SWCNTs, including commercially available lipases [180, 181]. Additionally, SWCNTs are inherently fluorescent materials in which changes in fluorescence can be used as a tool for analysis in enzymatic degradation. As an example, Kuo et al. electrostatically bonded the biodegradable anionic polyester-polyurethane blend, Impranil, which is industrially used in coating formulations, onto chitosan coated-SWCNTs [182]. When a free lipase from the fungus *Aspergillus oryzae*, known to hydrolyse Impranil, was added to a solution containing the Impranil/chitosan-coated SWCNTs, fluorescence emission at 1000 nm increased over 133 minutes. An increase in fluorescence emission was a result of enzymatic hydrolysis of the Impranil polymer by reducing the quenching effect of SWCNT electrostatic aggregation. Though beyond the scope of this review, it does highlight how the inherent properties of nanomaterials can be further applied to advance our knowledge in enzymatic polymer degradation [183].

Similar to SWCNTs, metal oxide NWs have found significant application in biomedical devices as potential biosensors and markers due to their inherent biocompatibility and unique optical properties [184, 185]. Consequently, enzyme immobilisation on the surface of metal oxide NWs has also been demonstrated, exploiting the conductive properties of the nanomaterial [185–189]. For example, Gkantzou and co-workers demonstrated that ZnO NWs can be employed as an immobilisation platform for β-glucosidase from the thermophile *Thermotoga maritima* [110]. In this study, polyethylenimine (PEI) was used as a capping agent to prevent enzyme leaching and reduce deactivation in an organic solvent. This resulted in retained activity in non-polar solvents including 86% and 91% in *n*-hexane and diisopropyl ether, respectively, after 24 h incubation at 50 °C. The researchers highlighted also the improved stability of the enzyme structure within this complex after repeated use, critical characteristics for sequentially testing polymer samples. However, the choice of metal oxide is crucial for each specific reaction. For example, metal ions such as Zn^2+^ and Fe^2+^ are α-amylase inhibitors, and as such, when iron oxide (FeO) NWs were used to immobilise an α-amylase from *A. niger* SAIB-4, starch degradation was inhibited and relative enzyme activity was reduced by 28% compared to when no metal was added [118]. Conversely, when copper oxide (CuO) NWs were added there was a 1.2-fold enhancement in activity with docking studies demonstrating that Cu^2+^ had a lower binding energy to the protein than Fe^2+^ allowing a lower energy barrier for the stable metal-protein complex to form.

Single dimensional nanomaterials exhibit high electronic conductivity due to their elongated structures providing the correct orientation to facilitate electron transport along the structure which limits the electronic resistance observed by O-D NPs [175]. Redox-dependent oxidoreductases immobilised to a 1-D nanomaterial can, therefore, benefit from an electrode in close proximity to improve the efficiency of metal porphyrin radical cation regeneration required for lignin oxidation. As an example, Liu et al. immobilised, by physical adsorption, the three extracellular lignin degrading enzymes; lignin and manganese peroxidase and the copper-containing laccase, extracted from a culture of the fungus *Cladosporium* sp. on SWCNTs [136]. The group demonstrated that all three enzymes had physically absorbed to the surface of the SWCNT and when the enzyme-NT complex was tested against a no SWCNT control for lignin degradation, observed a 12% increase in conversion over a 14-day time period. The improvement in activity, subsequently, was attributed to proximity of the enzymes to the electrode allowing for direct electron transfer to the metal-porphyrin core of each enzyme. As 1-D nanomaterials have become a promising target for use with enzymes with redox mechanisms multi-dimensional nanomaterials also exhibit these unique properties but also offer a diverse range of other benefits as enzyme immobilisation scaffolds which will be discussed in the next section.

### Multidimensional nanomaterials

Multidimensional nanomaterials conversely contain one or no dimensions at nanoscale, though for the letter the nanomaterial requires to be built through nanoscale components [190]. 2-D nanomaterials form a layered planar structure bonded together via van der Waals or electrostatic forces (Fig. 3C) [191]. For example, graphene is amongst the most studied 2-D nanomaterials organised into a hexagonal planar structure bonded through strong sp^2^ C–C bonds that form a continuous π-orbital cloud above and below the plane to allow a flow of electrons to pass between graphene layers [190]. Due to the chemical structure of graphene, the nanomaterial possesses unique chemical and physical properties as a result of electron mobility, enhanced mechanical strength and flexibility, as well as typically having a higher surface area (up to 2000 m^2^/g) than 1-D nanomaterials [192, 193]. When graphene is used as an enzyme scaffold, immobilisation commonly occurs onto the surface of the tightly layered nanostructure, providing further protection to the enzyme [194]. Though enzyme immobilisation can occur via weak hydrophobic interactions or electrostatic forces, the use of pure graphene as an enzyme immobilisation scaffold is limited and further functionalisation of graphene is required to allow for more stable covalent and non-covalent linkages and reduce hydrophobicity of the structure, limiting repulsive interactions with a typically hydrophilic enzyme surface [195]. Though examples of graphene decorated with organic functional groups as well as inorganic materials are prevalent, the most common method to functionalise graphene is by oxidation, using potassium permanganate and a strong acid, to prepare graphene oxide (GO) [196]. This chemical process partially breaks the aromaticity of graphene and provides hydroxyl and epoxide functional handles for enzyme immobilisation. GO, as a result, has become a immobilisation scaffold of interest for the study of PLF enzymatic degradation, with examples exhibiting the immobilisation of lipases, amylases and proteases to GO via covalent and non-covalent linkages [120, 132, 137]. It is worth noting that the disruption in the planar structure slightly compromises the electric conductivity of the original material, although this can be enhanced by further reduction to prepare a reduced form of GO, rGO, if enhanced electronic conductivity is required [197]. Conversely, metal oxide and transition metal dichalcogenides nanosheets are emerging as alternatives to graphene-based nanomaterials [198, 199]. The latter possess many similar physical and chemical properties to graphene by forming a 3-layer structure [200]. As a result, covalent immobilisation of β-amylase to molybdenum sulfide (MoS_2_) nanosheets (NS), using a glutaraldehyde linker, have been demonstrated [121]. Encouragingly, the enzyme retained 80% activity for starch degradation after ten uses, as well as, improving pH and temperature stability significantly. When using the free enzyme, retained activity was reduced by 70% when the pH was increased to pH 8.5 and temperature was increased to 90 °C, in separate experiments, but the group observed 80% and 70% retained activity, respectively, when the MoS_2_/β-amylase complex was used, highlighting its potential as an enzyme immobilisation scaffold. Though strong adsorption of the enzyme to the nanomaterial is desired, substrate adsorption to the nanomaterial surface needs to be considered. Khade et al., for example, immobilised a commercially available α-amylase onto a nanoporous ZnO nanosheet, via physical adsorption. [122] The researchers identified that the nanosheet forms an α-helix structure, immobilising both the enzyme and starch substrate which resulted in a reduction in maltose production. Despite the adsorption bringing both the substrate and biocatalyst in closer proximity, these observations suggests that this reaction is surface diffusion limited due to the propensity of the substrate to bind to the nanomaterial and a significant reduction in activity was observed.

One major drawback of using multidimensional nanomaterials is their synthesis and fabrication. The process can be time-consuming, costly, and use of finite resources. The use of alternative economically viable and accessible nanomaterials are of paramount importance, particularly, for the biodegradation testing industry which would require repeated synthesis and use of the nanomaterials [102]. Naturally occurring materials, such as pillared clay, have been classified as 2-D nanomaterials and potentially offer a sustainable enzyme immobilisation nanostructure [201]. Halloysite clay sheets are widely available and form aluminosilicate NTs with a hollow centre. This material contains a hollow lumen (diameter 15–20 nm), lined with aluminium hydroxide, which provides a protective barrier from environmental conditions, in addition to, the charge of the material remaining stable, in solution, between pH 3 and 10 [128, 201]. This allows for enzyme immobilisation control when the isoelectric point is known for the protein, as anionic proteins will form ionic linkages to the aluminium ions inside the NT and cationic proteins will immobilise to the negatively charged exterior, having being demonstrated with β-galactosidase, laccase and lipase enzymes [128, 202]. When targeting the interior of the aluminosilicate NTs, diffusion can influence the rate of degradation and, therefore, the diameter of the NTs lumen and hydrodynamic radius of the polymer substrate need to be considered, in addition to enzyme leaching. To overcome these issues, subsequent research on surface functionalisation has been carried out to improve scaffold-enzyme bond strength and introduce new physiochemical properties to the NTs’ [203]. Sillu and Agnihotri, for example, not only functionalised halloysite NT via aminosilanisation but also decorated the material with Fe_3_O_4_ NPs which introduced magnetic properties to the nanomaterial [111]. A cellulase mixture from *A. niger* was then covalently bound to the glutaraldehyde linkers and demonstrated an improvement in glucose production over 48 h from both lignocellulosic biomass derived cellulose and carboxymethylcellulose, compared to the reaction with the free enzyme over the same time frame, whilst the enzyme could be easily removed from the spent reaction media and reused.

Furthermore, 3-D interconnected porous nanostructures have also been employed as enzyme nanocarriers (Fig. 3D). Amongst the most applicable for enzyme immobilisation are silica based 3-D nanomaterials, due to their tunability of structure and pore size [204]. Modification of the silica structure can either be achieved during or post-synthesis to establish mesoporous silica nanoparticles (MSNs). A common method to synthesise MSN structures is with the use of a template compound, including cetyltrimethylammonium bromide (CTAB) and pluronic surfactants, which form 3-D structures such as micelles [205, 206]. The MSN is subsequently fabricated around this template before removal by calcination or extraction [207]. The choice of template is crucial in controlling pore size and nanomaterial structure and can be adapted for the application [208]. Hollow MSNs (HMSN), as an example, contain a cavity in the centre of the MSN which can be occupied with immobilised enzymes to provide enzyme protection [204, 205]. Another application demonstrated that the cutinase 2P from the yeast *Arxula adeninivorans* and lipase FE-01 from the fungus *Thermomyces languinosus* could be covalently immobilised onto the surface of a functionalised ellipsoidal HMSN shell containing a Fe_3_O_4_ NPs core. Not only did the presence of the magnetic NP improve recovery, an improvement in reaction turnover was also observed compared to the HMSN control reactions. Furthermore, hydrolysis of electrospun PCL fibres were observed using the lipase FE-01/HSMN/Fe_3_O_4_ complex highlighting its utility in polymer degradation [133]. Further common HMSN examples are from the Mobil Composition of Matter (MCM) and Santa Barbara Amorphous (SBA) series, which form solid silica nanostructures containing ordered mesopores differing in pore size and silica wall thickness which are used due to their ease of synthesis and thermal stability [209, 210]. The improved stability allowed Proteinase K from *Tritirachium album*, covalently immobilised in the pores of SBA-15 (diameter: 10.7 nm), to be extruded into poly(*L*-lactide) (PLLA) films to improve PLLA biodegradability [89]. Subsequently, the time taken to reach 100% biodegradability in compost reduced from 166 days, without SBA-15/ProK, to 106 days, highlighting the importance of the immobilisation of the enzyme on the nanomaterial towards improving stability of the enzyme to accelerate native biodegradation. Despite the examples highlighted, enzymatic activity and polymer diffusion within the 3D mesoporous silica structures is still restricted within the 3D structure and hydrolysis is often constrained to the materials surface if the materials pore size is not sufficient [211]. To respond to this constraint, dendritic or wrinkled MSNs have received attention due to their accessible structures which are synthesised using a cosolvent, i.e. pentanol or isopropanol in a micro-emulsion, to enhance the distance between each ‘wrinkle’ [206]. One limitation of these structures toward enzyme immobilisation is that functionalisation of the material for covalent bonding is difficult to control, therefore, literature examples commonly rely on physical absorption. As a result, there are multiple examples of the cellulase enzyme blends, which demonstrate a high affinity towards silica, bound to wrinkled MSN nanostructures which provide a protective environment for the enzymes without blocking substrate accessibility and, therefore, limiting the reduction in retained activity [40, 112]. Beyond silica-based 3-D nanostructures, inorganic-protein nanoflowers have also proven to be an intriguing alternative. The proteins form a cross-linked aggregate assembled around an inorganic centre including Cu_3_(PO_4_)_2_ [212]. Interestingly, Badoei-Dalfard et al. synthesised a nanoflower containing a cross-linked protein shell composed of collagenase from the *Bacillus* COL3 strain, surrounding an inorganic CuSO_4_/Fe_3_O_4_ nanoparticle core, which apart from improving the stability of the enzyme against temperature, pH and numerous organic solvents also enhanced activity for the degradation for collagen, albumin, gelatin, fibrin, and casein, highlighting its application for the study of the enzymatic degradation of protein-based PLFs [138].

### Enzymatic polymer degradation using continuous flow

An emerging area of research in recent years has been the combined use of continuous flow chemistry with biocatalysis [213–217]. An established method for conducting biocatalytic reactions on continuous flow requires the preparation an immobilised enzyme onto a fixed bed reactor enhancing the effective catalyst concentration within the bed reactor compared to a batch reaction [214, 217, 218]. The increased ability to control reactivity by managing flow rate, addition of the reagent and the removal of product, amongst other parameters, have often resulted cleaner reactions with improved yields and a reduction in reaction completion times [214, 219]. The benefits of continuous flow have resulted in more biocatalytic reactions being attempted in flow systems, leading to an increased number of publications in this area [215, 217]. One of the principal advantages of flow chemistry is the ability to conduct continuous reaction monitoring and analysis during the lifespan of the reaction. This is of particular benefit when determining reaction kinetics as well as the reaction mechanism [213, 217]. A drawback of current biodegradation studies is that the data output is limited to CO_2_ generation and O_2_ depletion. The determination of how the substrate is enzymatically degraded and subsequent by-product formation is currently inaccessible without solvent extraction of the inoculum in use, and only at the end of the test period. This knowledge is crucial in the development of new biodegradable polymers and determining whether toxic by-products are formed. Although continuous analysis can be performed in batch testing, a flow system with on-line (ultraviolet–visible (UV/Vis) spectroscopy, infrared (IR) spectroscopy, and (NMR) spectroscopy or at-line (mass spectrometry (MS), size exclusion (SEC) or high-performance liquid chromatography (HPLC)) continuous analysis can substantially increase the number of data points collected, which is amenable to artificial intelligence and machine learning approaches, while limiting the need to disrupt a closed system [213]. With the enhanced knowledge of how novel polymeric substances are broken down within the environment, potential environmental toxicity and persistence information could be obtained, and facilitate the further development and tuning of the polymer coming to market.

Current examples of use of hydrolytic and oxidoreductases enzymes within flow systems utilise inorganic enzyme supports or use entrapment within a sodium alginate matrix [220, 221]. However, the benefits of using an enzyme immobilised on a nanomaterial, outlined in this review, could provide unique benefits over the current standards within the field including the reduction of system back-pressure, due to the reduction in support material particle size, as well as, improved retained activity and reusability, crucial for repeated reliable analytical testing [222]. For example, Gal et al. prepared a fixed bed reactor containing lipase B from *C. antarctica* covalently immobilised onto SWCNTs for the kinetic resolution of a racemic mixture of 1-phenylethan-1-ol [129]. It was observed that the immobilised lipase remained active with use of vinyl acetate (VA) as an acyl donor over 4 weeks demonstrating highest productivity (2.04 product formation (μmol) min^−1^ mass of enzyme^−1^ (mg)) at 60 °C using 50 mg mL^−1^ *rac*−1-phenylethan-1-ol in hexane with a flow rate of 0.6 mL min^−1^. Though this example was not shown for polymer degradation, it highlights the immense potential of this technology for the continuous analysis polymer degradation of multiple samples without reactor replacement due to the stability of the enzyme over a 4-week period at elevated temperatures. In addition, the latest advancements in continuous flow biocatalysis performed on microreactors could prove crucial to the development of knowledge in enzymatic degradation mechanisms and potential by-products of the process [223]. Although this results in the reactions being performed at microscale, exploiting microchannels below 1 mm, reaction control is not compromised and key analytical biodegradation data can still be obtained [223]. Inspired by the use of an phenylalanine ammonia lyase from *Petroselinum crispum* immobilised onto magnetic NPs in a microchip, the kinetic resolution of *rac*−4-(morpholin-4-yl)butan-2-ol was performed in a U-shaped continuous flow microreactor using CALB immobilised onto silica coated Fe_3_O_4_ NPs positioned within a polytetrafluoroethylene tube (PTFE, internal diameter: 0.75 mm) [130, 224, 225]. The NPs were fixed into position using magnets and a mixture of the alcohol substrate and the acyl donor, VA, were pumped over 6 separate adjustable chambers at 1 µL min^−1^. Though the reaction was analysed through off-line gas chromatography (GC), continuous at-line GC analysis could be applied for more in-depth analytical data. Two plausible scenarios could arise when using continuous flow for the enzymatic degradation of PLFs substrates, i) low aqueous substrate solubility or ii) an increase in the viscosity of the reaction mixture, both of which could result in a back-pressure increase [226]. This could be mitigated by agitation to avoid solid particle build-up, though a more beneficial method could be to immobilise the biocatalyst to the surface of the reactor [217]. By coating the surface with functionalised nanomaterials, it provides an enhanced surface area for high enzyme loading and improved reactivity [113]. Microreactors are often used for reactor surface immobilisation to reduce the surface area-to volume ratio further and increase the effective enzyme concentration [223]. As an example, Gkantzou et al. continued their studies on enzyme immobilisation using ZnO NWs by immobilising the β-glucosidase from *T. maritima* to the NWs fabricated onto the wall of a silica microreactor, activated with PEI [110]. When comparing the hydrolysis of *p*-nitrophenyl β-D-glycopyranoside using a continuous flow system against the free enzyme there was not only a tenfold improvement in productivity (μg min^−1^ μg^−1^) but the reaction also required 26% less enzyme loading and half the reaction time for a 1.4-fold enhancement in product formation. When compared to the ZnO/PEI/β-glucosidase complex not within the continuous flow system there was a 30-fold improvement in productivity, highlighting the potential application using this system for enzymatic polymer degradation, as well as, more widely for the use of immobilised enzymes on nanomaterials in continuous flow systems to mitigate loss in overall activity. Even though this technology is in its infancy and further studies are required, it could have immense potential within PLF biodegradation analysis.

## Future outlooks

**Fig. 4:**
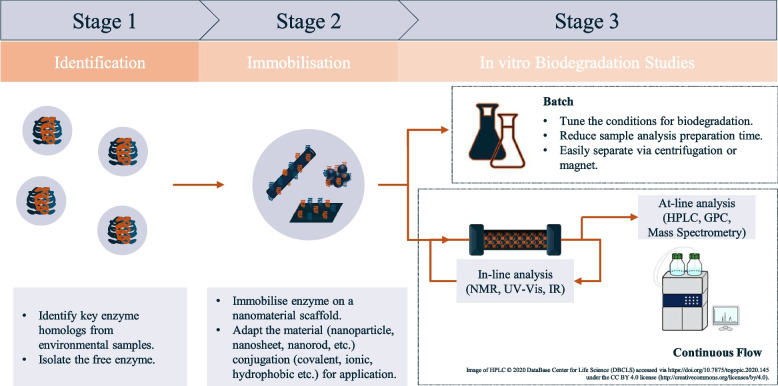
Proposed method to conduct enzymatic degradation studies of polymers, i) identify key enzyme homologs of environmental samples and isolate for in vitro testing, ii) immobilise isolated enzyme on nanoparticle/nanomaterial scaffold to improve reusability and performance, iii) conduct in vitro experiments either in batch or continuous flow allowing superior biodegradation data analysis to current methods

This review has highlighted the use and advantages of nanomaterials as enzyme scaffolds for the enzymatic degradation of various polymers classes including polysaccharides, polyesters, polyamides and lignin. These classes of polymers have been identified to be crucial in the ongoing development of the biodegradable PLF market. The development of this market hinges on the ability to control and assess biodegradation [1]. Current biodegradation tests are dependent on cell activity and viability and exhibit a low tolerance for deviating away from optimal growth conditions, therefore, biodegradation standard tests are limited by cellular function and growth [15, 95, 227]. Specifically, the biodegradation of polymeric substances is dependent on the extracellular secretion of enzymes from the microorganism to break down the polymer into monomeric units which subsequently can be metabolically assimilated [27]. Detailed analysis of the microbiome of different environmental samples is now possible and the enzymes required for polymer degradation are known, therefore, in vitro enzymatic degradation studies can now be carried out [228–231]. A potential method to perform these studies would be use a cell lysate of an environmental sample, however, this would present technical difficulties including complex sample analysis and the requirement to regulate metabolism and protein synthesis. Another solution would be to the isolate enzyme(s) known to carry out polymer degradation. Though recreating the complex nature of the microbiome of an environmental sample in vitro would be challenging, a representative sample set of vital isolated enzymes homologs could be formulated and compared against an environment control. Subsequently, the concentration of protein could be controlled and provide a specific environment for polymer degradation. Due to the lack of specific intracellular transport mechanisms, polymer biodegradation tends to proceed extracellularly and not in a tightly regulated intracellular environment [91]. In vitro studies allow for controlled degradation analysis and potentially accelerate the rates of depolymerisation for research and development purposes (Fig. 4). Furthermore, isolated enzymes could be immobilised onto a support scaffold. The advantages of using nanomaterials as an enzyme support scaffold are highlighted in this review and would mean polymer degradation studies could be carried out in a broader range of environmental conditions as well as reducing sample preparation time for analysis and enhancing reusability of the enzyme.

In addition, enzyme immobilisation has proven a useful tool for the combined use of biocatalysis and flow chemistry. One of the major advantages of the use of flow chemistry is the ability conduct continuous on-line or at-line testing using a wide range of analytical techniques including NMR, IR, MS and UV. Current biodegradation testing offers limited data output (CO_2_ generation or reduction in dissolved oxygen) and a continuous flow approach could provide continuous analysis of PLF degradation. In turn, further information about the mechanism of degradation and degradation products, vital for determining ecotoxicity and environmental fate, could be obtained. Though it would be difficult to alter international biodegradation standards using this technology alone, it is important to envisage and identify how new testing methods could shape polymer biodegradation studies in the future and how existing technologies can be repurposed to further the development of biodegradable polymers.

## Conclusions

Polymers in liquid formulation are a significant environmental pollutant and a key contributor to global microplastic formation. Therefore, it is vital that biodegradable polymers are identified and developed for use in the PLF market and recent regulation changes have reflected this. However, the development of novel biodegradable polymers is hindered by current biodegradation standard testing which is time limiting and offers limited data output. As a result, advanced biodegradation testing needs to be realised to improve turnaround times and feedback to further aid development. To address this, in vitro biodegradation studies would facilitate the use of multiple analytical techniques, and it would allow enzymatic polymer degradation to be analysed in isolation. Though testing could be carried out using the free enzyme in solution, this review has highlighted the advantages of using enzymes immobilised on nanomaterials for the degradation of common biodegradable polymers used in liquid formulation products. Nanomaterial scaffolds offer superior properties to conventional porous enzyme scaffolds and demonstrate improved enzyme activity, stability and robustness. The ability to carry out in vitro enzymatic degradation studies using immobilised enzymes could open a new way to analyse polymer degradation adopting alternative analytical techniques to assess reaction rates and biodegradation products. These advancements in degradation testing could be an invaluable screening tool, facilitate the development of more sustainable PLFs and speed up their pathway to market.

## Data Availability

No datasets were generated or analysed during the current study.
